# Clinical characteristics of extracorporeal cardiopulmonary resuscitation in China: a multicenter retrospective study

**DOI:** 10.1186/s12871-024-02618-2

**Published:** 2024-07-10

**Authors:** Zhiyong Yuan, Ying Liu, Guangyao Wei, Fuhua Wang, Bo Yao, Xiaotong Hou, Jinyan Xing

**Affiliations:** 1 Department of Critical Care Medicine, the Affiliated Hospital of Qingdao University, Qingdao, Shandong Province China; 2 Center for Cardiac Intensive Care, Beijing Anzhen Hospital, Capital Medical University, Beijing, 100029 China

**Keywords:** Extracorporeal cardiopulmonary resuscitation, Extracorporeal life support, Cardiac arrest, Epinephrine, Lactic acid, Neurological complications

## Abstract

**Purpose:**

Extracorporeal cardiopulmonary resuscitation (ECPR) might markedly increase the survival of selected patients with refractory cardiac arrest. But the application situation and indications remained unclear.

**Materials and methods:**

We respectively reviwed all adult patients who underwent ECPR from January 2017 to March 2021. Patient characteristics, initiation and management of ECMO, complications, and outcomes were collected and compared between the survivors and nonsurvivors. LASSO regression was used to screen risk factors. Multivariate logistic regression was performed with several parameters screened by LASSO regression.

**Results:**

Data were reported from 42 ECMO centers covering 19 provinces of China. A total of 648 patients were included in the study, including 491 (75.8%) males. There were 11 ECPR centers in 2017, and the number increased to 42 in 2020. The number of patients received ECPR increased from 33 in 2017 to 274 in 2020, and the survival rate increased from 24.2% to 33.6%. Neurological complications, renal replacement therapy, epinephrine dosage after ECMO, recovery of spontaneous circulation before ECMO, lactate clearance and shockable rhythm were risk factors independently associated with outcomes of whole process. Sex, recovery of spontaneous circulation before ECMO, lactate, shockable rhythm and causes of arrest were pre-ECMO risk factors independently affecting outcomes.

**Conclusions:**

From January 2017 to March 2021, the numbers of ECPR centers and cases in mainland China increased gradually over time, as well as the survival rate. Pre-ECMO risk factors, especially recovery of spontaneous circulation before ECMO, shockable rhythm and lactate, are as important as post-ECMO management,. Neurological complications are vital risk factors after ECMO that deserved close attention.

**Trial registration:**

NCT04158479, registered on 2019/11/08. https://clinicaltrials.gov/NCT04158479

**Supplementary Information:**

The online version contains supplementary material available at 10.1186/s12871-024-02618-2.

## Introduction

The overall survival rate of cardiac arrest (CA) patients receiving conventional cardiopulmonary resuscitation(CCPR) is between 10 and 30%, making it a global problem that seriously affects human health [1]. Failure to establish and maintain spontaneous circulation is the the main cause of high mortality and poor prognosis for CCPR. The application of venoarterial extracorporeal membrane oxygenation (VA-ECMO) has skyrocketed worldwide for adult patients in the last several years [2]. Under VA-ECMO, adequate organ perfusion is provided, blood pressure is sustained even during CA, making it a useful compensatory method for CCPR. The initiation of VA-ECMO in patients who are not salvageable by CCPR is termed extracorporeal cardiopulmonary resuscitation (ECPR). It includes the initiation of VA-ECMO after achieving ROSC with CCPR but sustaining shock status. Observational studies suggest that ECPR can increase the survival rate up to 30% for selected patients with refractory cardiac arrest of potentially reversible cause (e.g., myocardial infarction or pulmonary embolism) [3]. Data from the Extracorporeal Life Support Organization (ELSO) Registry have demonstrated an increase in annual ECPR cases from less than 100 in 2009 to more than 1500 in 2019 [4].

China is the most populous nation and the use of ECPR has increased lately. The number of ECPR cases and centers has increased yearly in mainland China. There has been no accurate or objective statistical data detailing the epidemiology of ECPR, especially the risk factors correlated with patient outcomes.

The Chinese Society of Extracorporeal Life Support (CSECLS) Registry Database is a voluntary registry, and pediatric and adult ECMO centers voluntarily upload information about the patient characteristics,information on the use, complications, and outcomes of supported patients in China [5]. ECMO is an expensive resource-intensive therapy,and its inappropriate application may result in a waste of resources. To improve the success rate of ECPR, screening and selecting proper patients ahead of ECPR initiation are important. Due to lack of strict criteria for patient selection, the high cost, and technical differences, prospective randomized controlled trials of ECPR are difficult to achieve. We conducted a retrospective analysis and screened all the available information of ECPR patients who were included in the database. This work provides a detailed nationwide epidemiological study of ECPR and its correlated risk factors based on real-world data in mainland China, in order to provide information to improve the use of ECPR.

## Materials and methods

### Study design and data collection

The CSECLS Registry Database from January 2017 to March 2021 was retrospectively reviewed. Data were collected using a standardized electronic reporting sheet submitted on the organization's website [5]. We queried the database for all adults cases (at least 18 years of age), which were diagnosed as a documented refractory cardiac arrest that occurred in the hospital or outside the hospital.

**Fig. 1:**
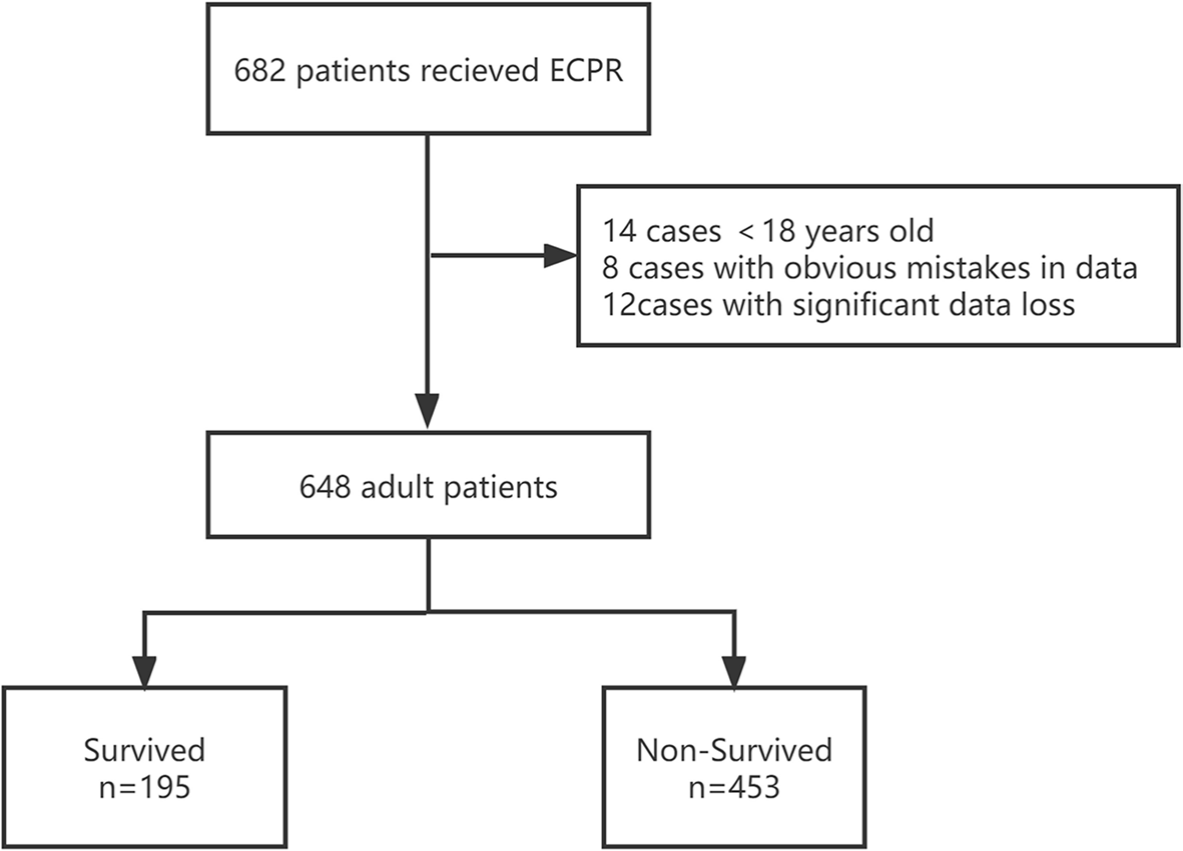
The flow chart of patient enrollment

The following data were collected: (1) Patient information:age, sex, body mass index (BMI), and comorbidities. (2) Resuscitation situations: CA location, primary cardiac rhythm, witnessed arrest, defibrillation, and return of spontaneous circulation (ROSC) before ECMO. (3) CPR process interventions:initiation and duration of CCPR and ECPR. (4) Post-ECPR treatments: mechanical ventilation, intra-aortic balloon pump (IABP), renal replacement therapy (RRT), targeted temperature management (TTM), vasoactive drugs, further operations and others. (5) Collected laboratory results: lactate, central venous oxygen saturation(ScvO2), hematokrit(HCT) before and after ECPR. Lactate clearance was defined by the equation of lactate clearance = [(lactate_*pre*_-lactate_*post*_)/ lactate_*pre*_] × 100%, where lactate_*pre*_ was the measured before ECMO and lactate_*post*_ was measured after 24 h of ECPR. (6) Patient outcomes: survival to hospital discharge or nonsurvival, duration of ECMO, IABP and mechanical ventilation, length of hospital stay and complications. The flow chart of patient enrollment is shown in Fig. 1.

Definitions of complications during ECMO: (1) Operating complications: Severe hemorrhage requiring surgery during manipulation including vascular dissection, retroperitoneal hematoma, damage to abdominal organs; air in circuit and decannulation. (2) Mechanical complications: Complications related to ECMO cannula and equipment including oxygenator or centrifugal pump dysfunction; pipeline or connector rupture; heat exchanger failure; catheter placement-related problem and thrombosis. (3) Neurological complications: Intracranial hemorrhage; acute ischemic stroke; cerebral infarction; seizure; hypoxic-ischemic brain injury and brain death, usually occur during or immediately after ECMO and diagnosed by symptom or imaging examination. (4) Hemorrhagic complications: Gastrointestinal hemorrhage; cannula site hemorrhage; surgical incision hemorrhage; hemolysis (free plasma hemoglobin > 50 mg/dL)) and disseminated intravascular coagulation(DIC). (5) Hepatorenal complications: Hyperbilirubinemia (direct bilirubin > 2 mg/dL; indirect bilirubin > 13 mg/dL; or total bilirubin > 15 mg/dL) or increased creatinine (> 3.0 mg/dL). (6) Limb complications: Distal limb ischemia; necrosis; compartment syndrome and vessel damage which may require fasciotomy or amputation in severe cases.

The Qingdao University Research Ethics Board reviewed and approved the study (QYFYWZLL27030). Informed consent was waived by the Qingdao University Research Ethics Board, and all the procedures followed the Helsinki Declaration of 1975. The study was registered at *clinicaltrials.gov.* with trial registration number NCT04158479.

### Statistical analysis

Normally distributed data are expressed as the mean and standard deviation and were compared using Student’s t test. Nonnormally distributed data are presented as the median and interquartile range (IQR) and were analyzed using the nonparametric Mann–Whitney U test. Categorical variables are expressed as numbers and percentages and were compared with the chi-square test or Fisher’s exact test. We used the least absolute shrinkage and selection operator (LASSO) logistic regression model to select the most useful prognostic risk factors for ECPR. Multivariate logistic regression analysis was performed with *p* < 0.05 in the univariate analysis as significant,as well as those screened as candidates by LASSO logistic regression, to find the independent risk factors for in-hospital mortality. The results are expressed as the *p* values and odds ratios (ORs) with the 95% confidence intervals (CIs). We used R software (version 3.6.1) to perform the LASSO logistic regression analysis. IBM SPSS 25.0 software was used for all the statistical analyses (IBM Corp., Armonk, NY, USA).

## Results

### Current situation of ECPR in China

Data were gathered from 42 ECMO centers covering 19 provinces in China. There were 11 ECPR centers in 2017, and increased to 42 in 2020. Only 1 center performed more than 10 ECPR procedures in 2017, and the number increased to 10 in 2020.

**Fig. 2:**
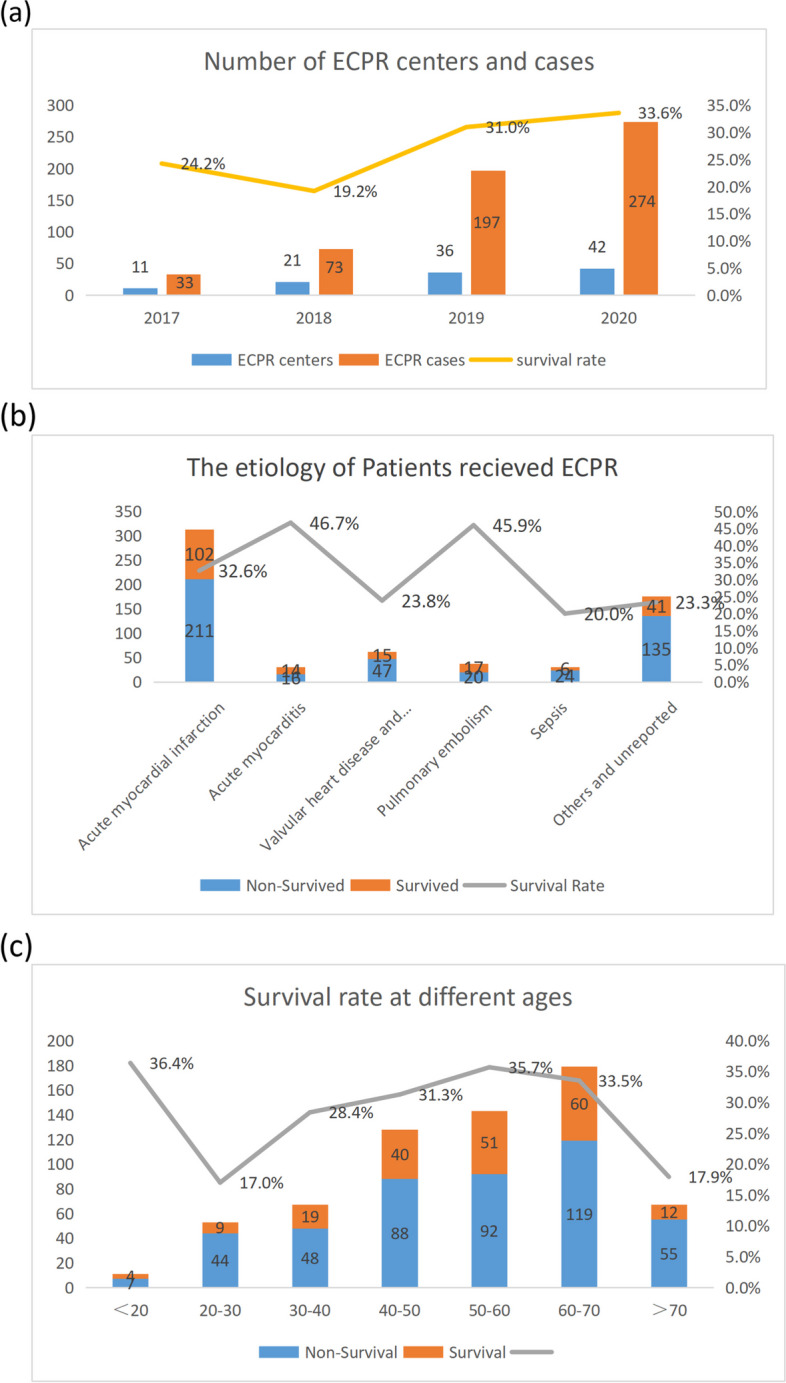
Trends in ECPR performance and outcomes in China. **a** Trends of ECPR centers and cases in China from 2017 to 2020. The number of centers and cases performed ECPR in mainland China has increased gradually over time, as well as the survival rate. **b** The etiology of patients underwent ECPR. Acute myocardial infarction was the most common, followed by aortic and valvular diseases. Acute myocarditis and pulmonary embolism were the causes of the highest survival (46.7% and 45.9% respectively), and sepsis was the cause of the lowest survival (20%). **c** Survival rate at different ages. Patients aged 20–30 years had the lowest survival rate. Then it increased slightly with age, and declined again after the age of 70

A total of 682 ECPR patients were screened from January 2017 to March 2021, and 648 patients were included in the study(Fig. 1). 491 (75.8%) patients were male. The average age was 53.38 ± 15.05 years. The number of patients who received ECPR gradually increased to 274 in 2020, 8.3 times than that in 2017. The survival rate was only 24.2% in 2017, and improved over years (19.2% in 2018, 31.0% in 2019 and 33.4% in 2020). Cardiogenic etiology accounted for 66.4% in ECPR patients, of which, acute myocardial infarction (72.8%) predominated. Patients with acute myocarditis had the highest survival rate (46.7%), and those with sepsis had the lowest survival rate (20%) (Fig. 2).

### Patient characteristics

Patient characteristics are shown in Table 1. 195 (30.1%) patients survived to hospital discharge, with a longer duration of ECMO than those nonsurvived (96.0 *vs.* 28.5 h, *p* < 0.001). Those survivors also had a longer duration of mechanical ventilation and a longer ICU stays and hospital stays than nonsurvivors, which might be due to survivor bias. Hemorrhagic complications were most common, occurrring in 143 cases (22.1%), followed by liver and kidney complications (76 cases, 11.7%), and nervous system complications (61 cases, 9.4%) (Table 2).

**Table 1:** Patient characteristics and interventions before ECMO

Variables	All *N* = 648	Non-Survived *N* = 453	Survived *N* = 195	*p*
Sex(male)	491(75.8%)	355(78.4%)	136(69.7%)	0.022
Age(year)	53.38 ± 15.05	53.30 ± 15.72	53.57 ± 13.40	0.826
BMI(kg/m^2^)	24.20 ± 3.64	24.35 ± 3.74	23.86 ± 3.39	0.123
ECPR Score	7.17 ± 4.55	6.96 ± 4.67	7.68 ± 4.19	0.217
Cardiac arrest location(OHCA)	188(29.0%)	139(30.7%)	49(25.1%)	0.357
By stander(yes)	539(83.2%)	376(83.0%)	163(83.6%)	0.710
Defibrillation(yes)	436(67.3%)	284(63.7%)	152(77.9%)	< 0.001
Primary disease				
Heart disease	430(66.4%)	290(64.0%)	140(71.8%)	0.493
Respiratory diseases	67(10.3%)	44(9.71%)	23(11.8%)	
other	151(23.3%)	119(26.3%)	32(16.4%)	
Cardiac rhythm				
Shockable rhythm	221(34.1%)	131(28.9%)	90(46.2%)	< 0.001
Non-Shockable rhythm	65(10.0%)	43(9.49%)	22(11.3%)	
Unclear	362(55.9%)	279(61.6%)	83(42.6%)	
Causes of arrests				
Acute myocardial infarction	313(48.3%)	211(46.8%)	102(52.3%)	0.008
Acute myocarditis	30(4.63%)	16(3.53%)	14(7.18%)	
Valvular heart disease and cardiomyopathy	63(9.72%)	47(10.38%)	15(7.69%)	
Pulmonary embolism	37(5.71%)	20(4.42%)	17(8.72%)	
Sepsis	30(4.63%)	24(5.30%)	6(3.08%)	
Others and unreported	176(27.2%)	135(29.8%)	41(21.0%)	
Comorbidities				
Hypertension	250(38.6%)	182(40.2%)	68(34.9%)	0.144
Diabetes	127(19.6%)	85(18.8%)	42(21.5%)	0.135
Respiratory diseases	24(3.70%)	20(4.41%)	4(2.05%)	0.158
Ischemic cardiac disease	69(10.6%)	51(11.3%)	18(9.23%)	0.134
Cardiac failure	63(9.72%)	44(9.71%)	19(9.74%)	0.808
Renal failure	20(3.09%)	17(3.75%)	3(1.54%)	0.216
Liver cirrhosis	6(0.93%)	4(0.88%)	2(1.03%)	0.711
Cerebrovascular disease	38(5.86%)	29(6.40%)	9(4.62%)	0.610
Malignant tumor	19(2.93%)	9(1.99%)	10(5.13%)	0.087
Smoking	196(30.2%)	138(30.5%)	58(29.7%)	0.966
ROSC before ECMO	110(17.0%)	58(12.8%)	52(26.7%)	< 0.001
Places of cannulation(in hospital)	580(89.5%)	409(90.3%)	171(87.7%)	0.612
Time interval of ROSC(min)	40(20,74)	45(24,75)	30(10,60)	0.665
Time from CA to CPR..(min)	1(0,5)	1(0,5)	1(0,2)	0.013
Time from CPR to ECMO.(min)	34(20,64)	39(20,65)	30(18,60)	0.050
Epinephrine before ECMO	269(41.5%)	203(44.8%)	66(33.8%)	0.012
Norepinephrine before ECMO	453(69.9%)	283(62.5%)	170(87.2%)	0.189
Pituitrin before ECMO	27(4.17%)	20(4.42%)	7(3.59%)	0.831
Dopamine before ECMO	210(32.4%)	155(34.2%)	55(28.2%)	0.144
Milrinone before ECMO	8(1.23%)	7(1.55%)	1(0.51%)	0.446
Epinephrine dosage before ECMO(µg/kg·min)	0.00(0.00,0.27)	0.00(0.00,0.50)	0.00(0.00,0.11)	0.001
Norepinephrine dosage before ECMO(µg/kg·min)	0.22(0.00,1.00)	0.30(0.00,1.08)	0.12(0.00,1.00)	0.048
Dopamine dosage before ECMO(µg/kg·min)	0.00(0.00,8.00)	0.00(0.00,8.00)	0.00(0.00,4.10)	0.101

*ECMO* Extracorporeal membrane oxygenation, *ECPR* Extracorporeal cardiopulmonary resuscitation, *ROSC* Return of spontaneous circulation

**Table 2:** During and post-ECPR treatments

Variables	All *N* = 648	Non-Survived *N* = 453	Survived *N* = 195	*p*
Time from ECMO team answering the phone to the bedside(min)	10(5,20)	10(5,20)	10(5,15)	0.568
Time from the initiation of ECMO to run (min)	27(20,40)	28(20,40)	25(20,39)	0.351
From ECMO team answering the phone to the initiation of ECMO(min)	40(30,60)	41(30,60)	40(30,58)	0.267
IABP	194(29.9%)	129(28.5%)	65(33.3%)	0.225
IABP duration	95.34(26.58,157.50)	56.75(19.00,121.00)	142.5(96.97,204.50)	0.001
Mechanical Ventilation	630(97.2%)	443(97.8%)	187(95.9%)	0.196
Mechanical Ventilation duration	67.98(21.58,180.42)	40.4(14.88,119.96)	168.50(92.08,277.45)	0.002
target temperature management	385(59.4%)	266(58.7%)	119(61.0%)	0.848
RRT	312(48.1%)	231(51.0%)	81(41.5%)	0.017
Operation after ECPR	59(9.10%)	37(8.17%)	22(11.3%)	0.419
Epinephrine after ECMO	183(28.2%)	133(29.4%)	50(25.6%)	0.344
Norepinephrine after ECMO	328(50.6%)	231(51.0%)	97(49.7%)	0.797
Pituitrin after ECMO	26(4.01%)	21(4.64%)	5(2.562%)	0.278
Dobutamine after ECMO	85(13.1%)	55(12.1%)	30(15.4%)	0.257
Milrinone after ECMO	5(0.78%)	3(0.66%)	2(1.020%)	0.640
Dopamine after ECMO	169(26.1%)	113(24.9%)	56(28.7%)	0.330
Epinephrine dosage after ECMO(µg/kg·min)	0.00(0.00,0.04)	0.00(0.00,0.06)	0.00(0.00,0.01)	0.067
Norepinephrine dosage after ECMO(µg/kg·min)	0.02(0.00,0.50)	0.02(0.00,0.80)	0.00(0.00,0.30)	0.040
ECMO duration(hour)	49.77(15.14,114.72)	28.5(9.98,88.27)	96.0(52.67,148.13)	< 0.001
Length of stay(day)	5.00(1.00,17.00)	3.00(1.00,8.00)	21.00(12.00,31.00)	< 0.001
Length of ICU stay(day)	4.00(1.00,11.00)	2.00(1.00,6.00)	13.00(7.00,19.00)	< 0.001
Operating complications	26(4.01%)	13(2.87%)	13(6.67%)	0.030
Mechanical complications	31(4.78%)	20(4.42%)	11(5.64%)	0.548
Hemorrhagic complications	143(22.1%)	108(23.8%)	35(17.9%)	0.100
Neurological complications	61(9.41%)	56(12.4%)	5(2.56%)	< 0.001
Hepatorenal complications	76(11.7%)	51(11.3%)	25(12.8%)	0.595
Limb complications	43(6.64%)	37(8.17%)	6(3.08%)	0.016

*ECMO* Extracorporeal membrane oxygenation, *ECPR* Extracorporeal cardiopulmonary resuscitation, *IABP* Intra-aortic balloon pump, *RRT* Renal replacement therapy

As for the characteristics before ECMO more survivors underwent defibrillation (77.9% *vs.* 62.7% *p* < *0.001*) and achieved ROSC before ECMO (26.7% *vs.* 12.8%, *p* < 0.001). Fewer survivors were given epinephrine (33.8% *vs.*44.8%, *p* = 0.012) and they went a shorter time from CA to CCPR and a shorter time from CPR to ECMO installation than nonsurvivors did. A total of 68 patients received ECMO outside hospitals. Among 580 patients who underwent with ECMO in hospitals, ICUs (264, 40.7%) and emergency departments(198, 30.6%) were the most common places for cannulation, followed by the catheterization room (55, 8.5%) and general wards(29, 4.5%).

Lactate was significantly higher in the nonsurvival group than in the survival group before installation of ECMO(13.04 ± 5.83 *vs.*10.62 ± 5.68, *p* < *0.001*). In As for the initiation and duration of ECPR, there were no differences in the time when the ECMO team answered the phone to the bedside between the two groups (*p* = 0.568), neither noror in the time from the initiation of ECMO to run.

In post-ECPR management, nonsurvival patients received higher doses of norepinephrine (1.00 *vs.* 0.58 µg/kg·min, *p* < *0.001*) and epinephrine (0.20 *vs.* 0.50 µg/kg·min, *p* = 0.004), with no difference in other vasoactive drugs. More patients in the nonsurvival group were treated with RRT than in the survival group (51.0% *vs.* 41.5%, *p* = 0.017). After 24 h of ECMO treatment, the lactate concentration was significantly lower in survival patients than those non-survived (3.62 ± 2.98 *vs.*8.70 ± 6.33, *p* < *0.001*), and the lactate clearance rate was higher (71.1%,IQ (50.0%,84.2%) *vs.* 42.50%, IQ (-0.94%,68.5%), *p* < *0.001*) (Table 3). The survivors had more neurological complications and limb complications than the nonsurvivors (12.4% *vs.*2.6%, *p* < *0.001* and 8.2% *vs.*3.1%, *p* = 0.016 respectively).

**Table 3:** Collected laboratory results

Variables		All *N* = 648	Non-Survived *N* = 453	Survived *N* = 195	*p*
Before installation of ECMO	Lactate(mmol/L)	12.28 ± 5.88	13.04 ± 5.83	10.62 ± 5.68	< 0.001
	ScvO2(%)	52.40(40.00,67.40)	50.50(40.00,67.40)	55.00(40.00,67.00)	0.407
	HCT(%)	36.27 ± 10.53	36.26 ± 11.03	36.31 ± 9.23	0.785
24 h after ECMO	Lactate(mmol/L)	6.65 ± 5.80	8.70 ± 6.33	3.62 ± 2.98	< 0.001
	ScvO2(%)	69.72 ± 15.1	69.24 ± 15.69	70.38 ± 14.35	0.849
	Lactate clearance(%)	54.4%(8.97%,76.3%)	42.50%(-0.94%,68.5%)	71.1%(50.0%,84.2%)	< 0.001

Lactate clearance = [(lactate_*pre*_ − lactate_*post*_)/ lactate_*pre*_]*100%. Lactate_*pre*_ was measured before ECMO and lactate_*post*_ was measured after 24 h of ECPR

*ScvO2* Central venous oxygen saturation, *HCT* hematokrit

### LASSO regression and multivariate logistic regression analysis

LASSO regression was used to screen parameters, and the variation characteristics of coefficienst of these variables are shown in Supplemental Fig. 1. The tenfold cross-validation method was applied to the iterative analysis, and a model with excellent performance but a minimum number of variables was obtained when λ was 0.04 (log λ = -3.2).

**Fig. 3:**
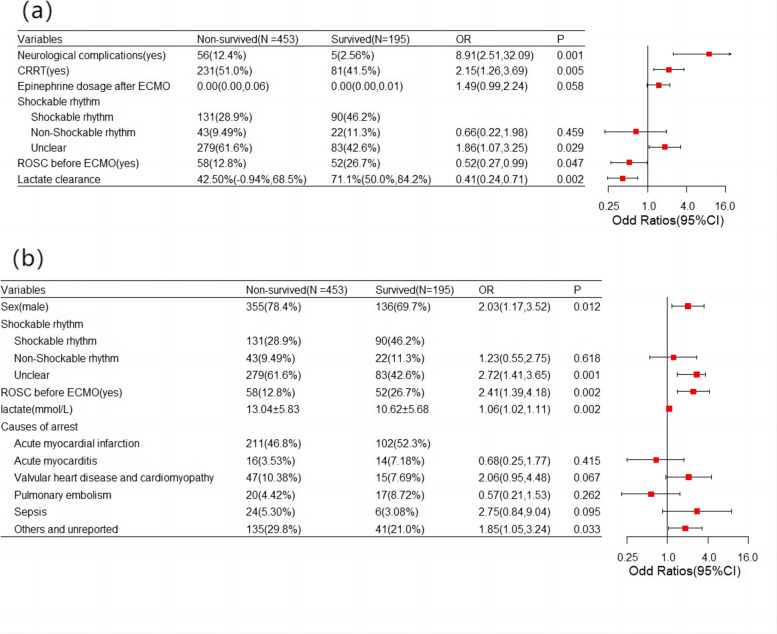
Multivariable analysis conducted to identify independent risk factors affecting patient outcomes during the whole process and before the initiation of ECMO. **a** ROSC before ECMO, neurological complications, epinephrine dosage after ECMO, CRRT and lactate clearance were risk factors independently associated with ECMO outcomes. **b** Sex, recovery of spontaneous circulation ROSC before ECMO, causes of arrest, shockable rhythm and lactate before installation of ECMO were characteristics before initiation of ECMO that independently affected the outcome

Multivariable analysis was conducted to identify independent risk factors affecting patient outcomes. The presence of ROSC before ECMO, neurological complications, epinephrine dosage after ECMO, use of CRRT and lactate clearance were risk factors independently associated with the outcome (Fig. 3a). Among characteristics before the initiation of ECMO, sex, recovery of spontaneous circulation ROSC before ECMO, cause of arrest, shockable rhythm and lactate before the installation of ECMO were risk factors independently affecting the outcome (Fig. 3b).

## Discussion

The present retrospective study aimed to assess the effectiveness of ECPR in China. Through a nationwide ECPR survey, we found that ECPR is widely carried out in China, with more ECMO centers involved, and an increasing number of patients with severe conditions might benefit from ECPR.

The survival rate of patients who underwent ECPR was 30.1% in our study, which is significantly higher than the reported 22% survival rate for in-hospital cardiac arrest and the less than 15% for out-of-hospital cardiac arrest [6]. Although the selection bias may be strong, a percentage of patients who fail to respond to CCPR could benefit from ECPR. According to the ELSO Live Registry Dashboard in the ELSO registry updated through February 2022, 11443 cases of ECPR in adult patients were reported worldwide, with 29% surviving to DC or transfer. Our results are consistent with these reports.

Since 2017, the ECPR survival rate in China has been gradually increasing, reaching 24.2% in 2017, and increasing to 33.4% in 2020, which may be related to the modification of technology, the control of complications, and the rising popularity of CPR.

Analysis of risk factors showed that the need for epinephrine and higher lactate levels were associated with poor outcomes both before and after ECPR. Neurological complications play a vital role in patient survival in post-ECMO management.

Acute myocardial infarction is the dominant cause of ECPR and is more common in elderly individuals. Patients with myocarditis and pulmonary embolism had the highest survival rate, at 46.7% and 45.9% respectively. This may be due to the reversibility of heart function. With ECMO to ensure tissue perfusion, cardiac function can be restored after primary disease treatment. In contrast, patients with severe infection and sepsis had the lowest survival rate of 20.0%. Severe inflammatory response in sepsis patients combined with ECMO-induced cascade of inflammatory responses exacerbates multisystem organ failure, which may be an important factor in their poor survival [7]. Furthermore, patients over 70 years old and those under 40 years old had the lowest survival rates, at 17.9% and 24.4%, respectively. Patients over 70 years old might suffer from more comorbidities and poor basic health conditions. Patients under 40 years of age had an even higher mortality rate, probably because of the high proportion of other causes and unknown causes, including trauma and asphyxia of other causes, most of which are irreversible or difficult to correct.

Lactate and lactate clearance were associated with patient outcomes. The arterial blood lactate level, a reflection of the body’s metabolic level, has a strong correlation with prognosis and is known to be associated with mortality in sepsis and other forms of critical illness [8]. Lactate clearance reflects the change in lactate concentration at different times and represents an accessible method for assessing tissue oxygen delivery. The increase in lactic acid levels is mainly due to ischemia and hypoxia, and once this situation is corrected, lactic acid levels begin to decrease. A 20% reduction in lactic acid concentration had been proved to improve patient outcomes [9]. Moreover, lactate levels can be elevated by other factors, such as liver dysfunction, which can seriously affect patient outcomes [10]. An increase in blood lactate after several hours of ECMO support could be indicative of a severe complication (e.g. mesenteric ischemia) to be addressed immediately.

Epinephrine has been the cornerstone of cardiac resuscitation because of its action of increasing coronary and cerebral perfusion pressure through stimulation of α1 receptors in vascular smooth muscle causes vasoconstriction [11]. However, some recent studies have begun to question the role of epinephrine in cardiac arrest patients and reported no improvement in survival to hospital discharge or survival with favorable neurological outcomes after OHCA [12, 13]. There have been no randomized controlled trials evaluating epinephrine therapy in humans in the context of VA-ECMO. In our study, the more epinephrine was administered after ECMO, the worse the prognosis of patients. Consistent with Nesseler's findings, among patients who required VA-ECMO, epinephrine administration was associated with an increased risk for death [14]. Several mechanisms may explain the worse outcomes observed. First, epinephrine increases both cardiac output and blood pressure via its beta-adrenergic effect, while at the same time, contributing to the development of arrhythmias, increased myocardial oxygen consumption and unfavorable metabolic effects [15]. Epinephrine might increase lactate levels by pyruvate generation through a cAMP-dependent mechanism and increase cardiac double products in cardiogenic shock patients [8]. Second,epinephrine represses drug metabolism enzymes and induces a local inflammatory response via interleukin-6 production in vitro cell assays [16]. Finally, the use of epinephrine is heavily biased by severity of arrest/shock. It is difficult to control for bias between resuscitation time and epinephrine use. Peripheral cannulation for VA-ECMO increases left ventricular (LV) afterload, often leading to increased LV end-diastolic pressure and decreased stroke volume. Inotropes especially epinephrine, are used to enhance LV ejection and prevent LV distension [17]. A higher epinephrine dosage indicates poorer cardiac function. IABP may be an alternative strategy, although studies reported no significant improvement in survival with the concurrent use of IABP and VA-ECMO for cardiogenic shock [18]. Our study had similar results, but we found that the use of epinephrine was reduced in IABP patients (0.12 ± 0.39 in IABP patients *vs.*0.33 ± 1.50 in non-IABP patients), which may improve the prognosis of patients in another way. More prospective multicenter randomized trials on epinephrine use are needed to verify the safety and efficacy of epinephrine in patients receiving ECMO.

Neurological complications negatively affect patient mortality and quality of life. Neurological complications mainly include hypoxic-ischemic encephalopathy, cerebral infarction, cerebral hemorrhage, and even vegetative state or brain death. The incidence of neurological complications was 9.3% in our investigation, which was lower than that in other reports [19]. This might be because neurological function was not assessed in patients who were dying, and it was difficult to detect mild neurological impairment in asymptomatic patients. Limb complications mainly include distal ischemia, bleeding, dissection and compartment syndrome [20]. The occurrence of neurological and limb complications may be related to comorbidities, anticoagulation management, and the correction of ischemia and hypoxia [21, 22]. Early detection of neurological and limb complications is important for immediate action to avoid severe consequences.

## Conclusion

From January 2017 to March 2021, the number of centers and patients who underwent ECPR in mainland China increased gradually over time, as well as the survival rate. Pre-ECMO risk factors, especially the need for epinephrine, the lactate level before ECMO and lactate clearance after ECMO installation, are as important as post-ECMO management. Neurological complications are vital risk factors after ECMO and deserve close attention.

### Supplementary Information

Supplementary Material 1.

## Data Availability

The data are available from the corresponding author on reasonable request.
