# Supplementary figures and images for: Clinical characteristics of extracorporeal cardiopulmonary resuscitation in China: a multicenter retrospective study
Source: BMC Anesthesiol. 2024 Jul 10;24:230. doi: 10.1186/s12871-024-02618-2 (PMC11234634; doi:10.1186/s12871-024-02618-2)

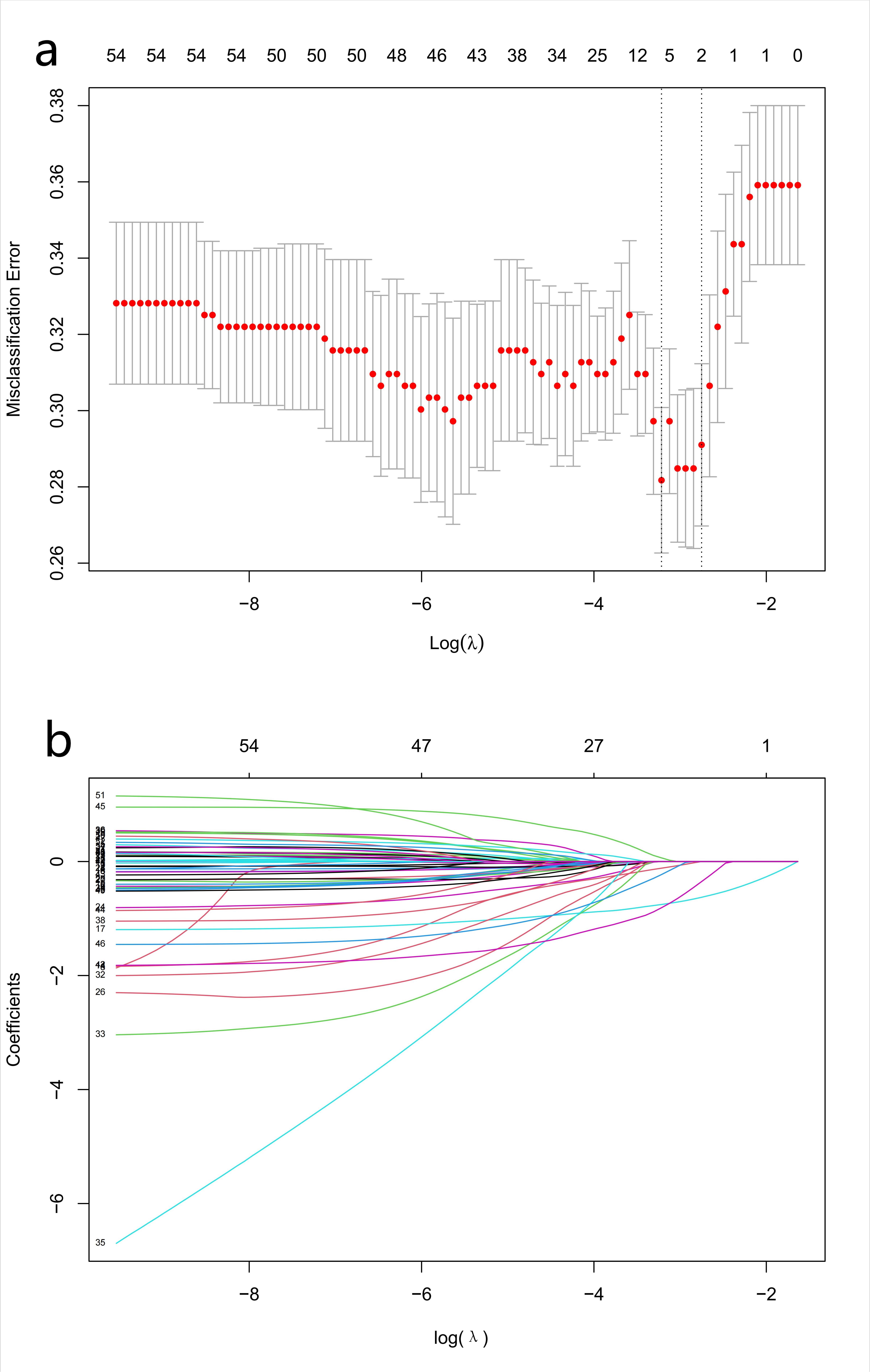

Supplement: Supplementary file 1 — Supplementary Material 1. [file 12871_2024_2618_MOESM1_ESM.jpg]
